# Using extracellular matrix derived from sugen-chronic hypoxia lung tissue to study pulmonary arterial hypertension

**DOI:** 10.3389/fphar.2023.1192798

**Published:** 2023-09-05

**Authors:** Patrick A. Link, Laszlo Farkas, Rebecca L. Heise

**Affiliations:** ^1^ Department of Biomedical Engineering, Virginia Commonwealth University, Richmond, VA, United States; ^2^ Division of Pulmonary, Critical Care and Sleep Medicine, Department of Internal Medicine, Davis Heart and Lung Research Institute, College of Medicine, The Ohio State University Wexner Medical Center, Columbus, OH, United States

**Keywords:** pulmonary arterial hypertension, substrate stiffness, ECM, angiogenesis, decellularization

## Abstract

Pulmonary arterial hypertension has characteristic changes to the mechanical environment, extracellular matrix, and cellular proliferation. In order to develop a culture system to investigate extracellular matrix (ECM) compositional-dependent changes in pulmonary arterial hypertension, we decellularized and characterized protein and lipid profiles from healthy and Sugen-Chronic Hypoxia rat lungs. Significant changes in lipid profiles were observed in intact Sugen-Hypoxia lungs compared with healthy controls. Decellularized lung matrix retained lipids in measurable quantities in both healthy and Sugen-Chronic Hypoxia samples. Proteomics revealed significantly changed proteins associated with pulmonary arterial hypertension in the decellularized Sugen-Chronic Hypoxia lung ECM. We then investigated the potential role of healthy vs. Sugen-Chronic Hypoxia ECM with controlled substrate stiffness to determine if the ECM composition regulated endothelial cell morphology and phenotype. CD117+ rat lung endothelial cell clones were plated on the variable stiffness gels and cellular proliferation, morphology, and gene expression were quantified. Sugen-Chronic Hypoxia ECM on healthy stiffness gels produced significant changes in cellular gene expression levels of Bmp2, Col1α1, Col3α1 and Fn1. The signaling and cell morphology observed at low substrate stiffness suggests early changes to the ECM composition can initiate processes associated with disease progression. These data suggest that Sugen-Chronic Hypoxia ECM can be used to investigate cell-ECM interactions relevant to pulmonary arterial hypertension.

## 1 Introduction

In Pulmonary Arterial Hypertension (PAH), many factors contribute to the progressive and irreversible nature of the disease including altered mechanical environment, increased extracellular matrix (ECM) deposition, apoptosis, proliferation, and cellular contraction (Stenmark et al., 2016; Karki and Birukova, 2018; Wang et al., 2019). Many groups have shown a role for ECM on disease progression in other lung diseases (Loessner et al., 2010; Booth et al., 2012; Sokocevic et al., 2013; Scarritt et al., 2014; Wagner et al., 2014; Sava et al., 2017). One strategy to study the contribution of the ECM to hypertensive disease progression used decellularized rat lungs seeded with naïve mesenchymal stem cells to show a persistence of the hypertensive phenotype in rat models (Scarritt et al., 2014). However, this study did not identify the contribution of the ECM versus the mechanical environment alone to the persistence of the hypertensive hallmarks.

Extracellular matrix (ECM) is all the macromolecules present outside of the cell. These molecules can be lipids, proteins, glycoproteins, and polysaccharides. The ECM provides structural support for cells, allowing cells to attach, preventing cell movement, or providing a track to promote migration. The ECM is also a primary source of signal initiation. ECM can bind and release growth factors, transmit mechanical signaling forces through integrins, or begin signaling pathways using fragments of broken down ECM (Lu et al., 2012). Macromolecules, like fibronectin, collagen, tenascin-c, and sphingosine-1 phosphate generally increase in PAH (Bu et al., 2008; Jia et al., 2017). In PAH, the changes in ECM lead to changes in the structural micro-niches. Such structural changes often come with characteristic changes to the mechanical properties: more collagen leads generally to increased stiffness. However, groups have not decoupled the experiments to determine if compositional make-up can alter cellular phenotype independent of mechanical changes.

Elucidating the composition-induced effects on cellular behavior in lung diseases like PAH are critically important. To date, no one knows whether ECM composition alone is capable of inducing cellular disfunction in PAH. However, cytokines and hormone have half-lives on the order of minutes to hours, whereas proteins have half-lives on the order of days (Rahman and Sadygov, 2017). Furthermore, most ECM studies consider only the structural components of the ECM like collagen or elastin rather than other bound signaling components within the matrix like sphingolipids. Sphingolipids are known to play both pro- and anti-inflammatory roles in atherosclerosis (Piccoli et al., 2023) and PAH (Chen et al., 2014). Both structural and signaling components of ECM arecritical to consider when studying to disease progression. As an initial model for investigating diseased ECM–cell interactions in PAH, we test ECM composition independent of substrate stiffness to identify the role each plays in progressing endothelial cell disease phenotype. We hypothesize that incorporating diseased ECM composition from Sugen-Chronic Hypoxia (Su/CHx) rats into mechanical experiments is capable of producing the endothelial cell phenotype of disease.

## 2 Materials and methods

### 2.1 Decellularizing lung tissue

We decellularized lung tissue based on the same procedure described in (Link et al., 2017). We obtained healthy and Su/CHx left lung lobes from rats from a prior study (Bhagwani et al., 2019). We carefully dissected away large airways and vessels and then minced the distal lung tissue into small pieces to maximize the surface area for decellularization while minimizing the diffusion distance. We rinsed the tissue three times with PBS between each step. We submerged the lung tissue in 0.1% Triton X-100 solution for 24 h at 4°C, followed by 2% sodium deoxycholate for 24 h at 4°C. We drew the sodium deoxycholate out of the tissue by submerging the tissue in filtered NaCl solution for 1 h at 4°C. We removed the NaCl from the tissue with three rinses of ultrapure water (UPW). Then to remove the DNA, we submerged the tissue in filtered DNase solution for 1 h at 4°C, followed by three rinses with 1x PBS.

After decellularization, we lyophilized and cryomilled the tissue, to form a powder. We acid digested 10 mg of the powder in 1 mL of 0.01 M HCl with 1 mg of pepsin, under constant agitation, at room temperature for 4 h. We neutralized the acid with 0.1 M NaOH at a 1:10 ratio, and stored the solution at −80°C.

### 2.2 Protein differences in diseased ECM

After decellularization, before lyophilization, we took intact and decellularized tissues for quantitative mass spectrometry. We placed approximately 10 mg of tissue with PBS into a BeadBug tissue homogenizer. The tissue was homogenized three times for 30 s each time, vortexing in between homogenization. 100 mM ammonium bicarbonate containing a Rapigest concentration of 0.1%, 5 mL of 10 mM dithiothreitol in 0.1 M ammonium bicarbonate were added at room temperature for 0.5 h. Then 5 mL 50 mM iodoacetamide in 0.1 M ammonium bicarbonate was added at room temperature for 0.5 h. The samples were digested with 1 mg trypsin twice overnight and then quenched with 5% (v:v) glacial acetic acid. Each sample was analyzed in triplicate.

The samples were analyzed by a Waters Synapt G2Si mass spectrometer system with a nanospray ion source interfaced to a Waters M-Class C18 reversed-phase capillary column. Peptides were eluted using acetonitrile/0.1% formic acid gradient, with lockspray compound at a ow rate of 0.4 mL/min at 3.5 kV.

The data were analyzed by database searching using the PLGS search algorithm against the NCBIs rat database. Relative quantification was performed using the Progenesis program.

### 2.3 Sphingolipid differences in diseased ECM

To quantify differences in lipid content we used intact tissue and decellularized tissue. For the decellularized tissue, after decellularization, before lyophilization, we took intact and decellularized tissues for quantitative mass spectrometry. We placed approximately 10 mg of snap frozen tissue from each lung sample into a cryogrinder. Lipid levels were evaluated in the lung tissue by reverse-phase high-performance liquid chromatography separation, negative-ion electrospray ionization, and tandem mass spectrometry analysis, as previously described (Hait et al., 2009).

### 2.4 Cell culture and ECM stiffness experiments

Healthy and diseased ECM covalently bonded to 1 kilopascal (kPa) and 20 kPa polyacrylamide gels (PAGs) overnight. This established two expected environments 1 kPa +healthy ECM and 20 kPa +Su/CHx ECM, and two unusual environments 1 kPa +Su/CHx ECM and 20 kPa +healthy ECM. To determine if the results could be due to ECM protein adherence to the PAG, we quantified protein adsorption using a BCA (Pierce) assay.

CD117+ endothelial cell (EC) clones were isolated and cultured as previously published (Farkas et al., 2019). We added 20,000 CD117+ EC/cm^2^ (Bhagwani et al., 2019; Farkas et al., 2019) to the PAGs and allowed them to attach for 24 h. After 24 h, we changed the media until the timepoint indicated. To quantify proliferation, we used cell counting kit-8 (CCK-8; Dojindo) according to manufacturer’s directions.

### 2.5 Phenotype shifts measured through qPCR mRNA quantification

In brief, mRNA was isolated using a Qiagen mRNeasy kit protocol. Before converting to cDNA using iScript (BioRad) we balanced the mRNA to 25 ng/mL. Then we combined 4 mL cDNA with a mastermix consisting of individual forward and reverse primers (Supplementary Table S1), and SYBR green in a PCR plate. We covered the plate, placed it on a plate shaker for 2 min and centrifuged at 1,200 RPM for 2 min. To run qPCR, we placed the plate on a CFX Connect Real-Time System (BioRad) for thermocycling. We set the cycles to 95°C for 15 s, 58°C for 30 s, and then 72°C for 15 s to promote denaturing, primer annealing, and extension, respectively.

### 2.6 Statistics

Statistical analyses are reported in the figure captions. We determined statistical significance at *p* <0.05, using GraphPad Prism version 10.0.0 for Windows, GraphPad Software, Boston, Massachusetts USA, www.graphpad.com. All experiments were performed in n ≥3 experiments with technical replicates unless otherwise noted in the figure caption. Data presented mean ± SEM.

## 3 Results

### 3.1 Compositional differences in diseased ECM

**FIGURE 1:**
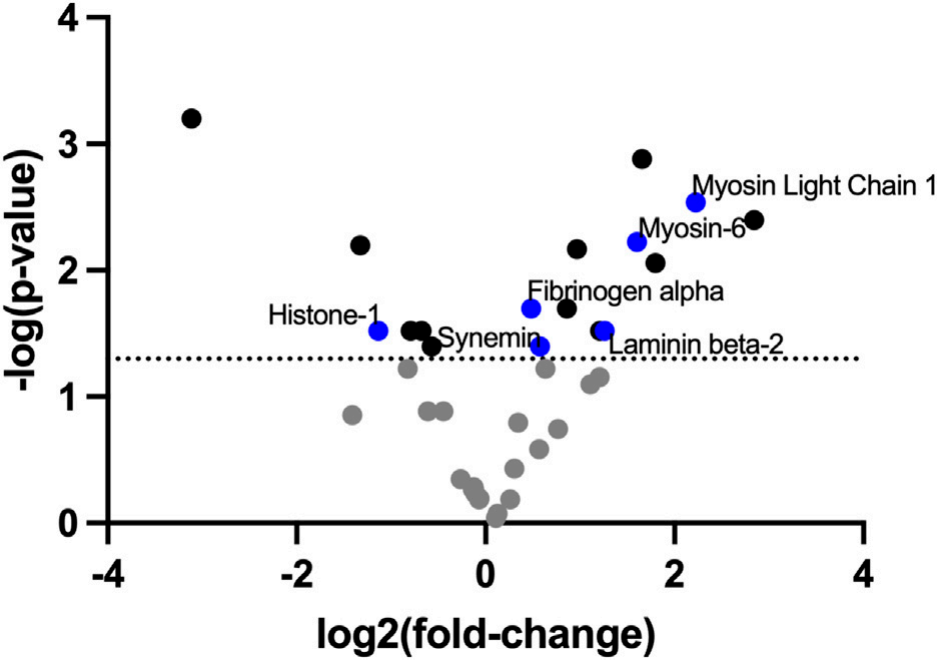
ECM decellularized from Su/CHx maintains significant differences compared to healthy tissue. Fold change presented as Su/CHx over healthy. Data points are averaged from three independent biological replicates (*n* = 3) in each group.

To identify the ECM compositional changes which occur in PAH, we performed mass spectrometry on decellularized healthy and Su/CHx lung tissue. Many proteins and lipids differences found through mass spectrometry of PAH lungs were also present in our Su/CHx tissue (Figure 1; Supplementary Table S2). In similar fold changes to our results, Myosin light chain, Myosin-6, Laminin Beta-2, Synemin, Fibrinogen α, Histone-1 have been shown upregulated in PAH as well (Suntharalingam et al., 2008; Huang et al., 2018). In comparing intact tissue to decellularized Su/CHx tissue, we found increased the presence of Laminin C and Protein RGD, and further increased the presence of Myosin-6 and Keratin-78, but decreased the presence of β-actin, which was increased in the intact Su/CHx tissue (Supplementary Table S3). Combined these intracellular and extracellular protein changes may provide clues to ECM compositional changes which continue to progress cellular disease phenotype.

**FIGURE 2:**
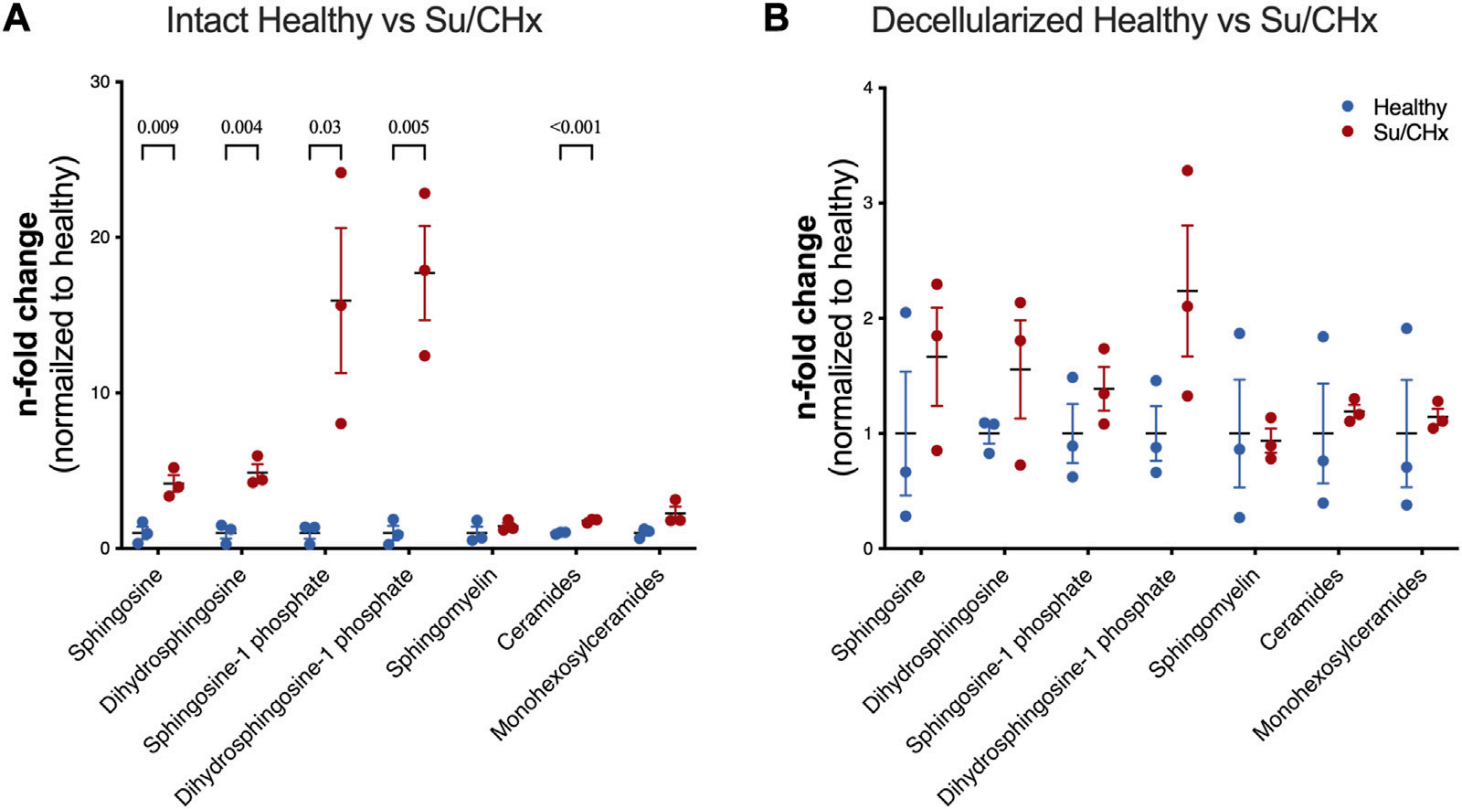
Lipids are increased in Su/CHx lung tissue. Differences between healthy and diseased lungs. Subspecies of sphingomyelins, ceramides, and monohexosylceramides have been normalized individually. **(A)** Lipid differences between intact healthy and intact Su/CHx lungs. **(B)** Lipid differences are decreased, but largely retained after decellularization. *N* = 3 from unpaired *t*-test, *p*-values line indicates *p* < 0.01, between groups as indicated. Data presented are individual biological replicates (points), mean, and SEM.

Decellularization is meant to remove lipids, specifically cellular membranes from tissue. However, lipids have been shown to play a role in PAH. S1P-induced a TGF-ß-like fibrosis but could be limited by Dihydrosphingosine-1 phosphate (Bu et al., 2008). Lipid analysis of intact healthy and Su/CHx tissues shows increased sphingosine, dihydrosphingosine, sphingosine-1 phosphate (S1P), dihydrosphingosine-1 phosphate (dHS1P), and ceramides with minimal changes to other lipids (Figure 2A). As expected, decellularization removes many of those lipids (Figure 2B), but the overall trend of differences was similar in decellularized Su/CHx vs. healthy lungs.

### 3.2 Decellularized protein solution as an *in vitro* coating

**FIGURE 3:**
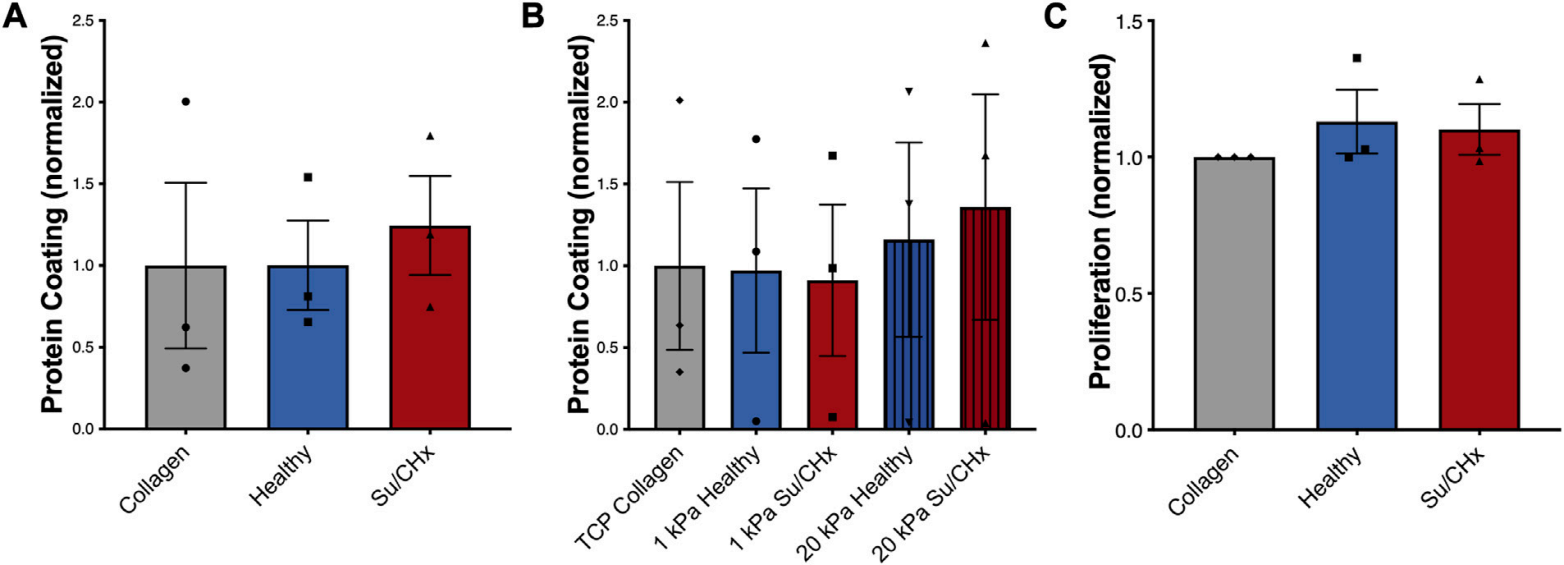
Su/CHx ECM does not affect CD117+ ECs proliferation. **(A)** Adsorption of heterogenous ECM protein is similar to collagen coating on TCP. *n* = 3; **(B)** Adsorption of heterogenous ECM protein is slightly increased on 20 kPa PAGs, though no statistically significant changes are observed. ECM coating measured through BCA. *n* = 3; **(C)** CD117+ ECs plated on TCP coated with collagen, healthy matrix, and Su/CHx matrix have statistically similar proliferation (Measured by CCK-8) after 24 h. *N* = 3 bars display mean ± SEM.

We first wanted to make sure that inherent differences between collagen, healthy ECM, and Su/CHx ECM did not have baseline differences as a coating, which could possibly affect cellular attachment and therefore cell behavior. To accomplish this, we first quantified how much protein adsorbed to the PAG. We added ECM to non-tissue treated plates overnight, rinsed twice with PBS, and then performed a BCA analysis on the adsorbed protein (Figure 3A). There was no significant difference in adsorbed protein between collagen, healthy ECM, and Su/CHx ECM coatings. We used a concentration of 0.05 mg/mL for all protein coatings because 0.05 mg/mL was shown to be the optimal concentration for cell attachment and proliferation (Zhang et al., 2009). Coating tissue culture plastic (TCP) and PAGs with 0.05 mg/mL protein produced similar coating efficacy compared to collagen on the same surface (Figure 3B).

### 3.3 CD117+ EC response to diseased ECM

#### 3.3.1 Cell proliferation and morphology on different ECMs

To determine if our results could be due to proliferation, we plated CD117+ ECs on different ECMs on tissue culture plastic and on 1 or 20 kPa poly-acrylamide gels coated with the various ECMs. On TCP, cell proliferation was not significantly different between ECM groups (Figure 3C). Similarly, on different stiffnesses, increased stiffness produced a slight increase in proliferation, but there were no significant differences (Supplementary Figure S1A). The Su/CHx tissue produced a slight decrease in proliferation on both 1 and 20 kPa PAGs, but the differences were not significant.

**FIGURE 4:**
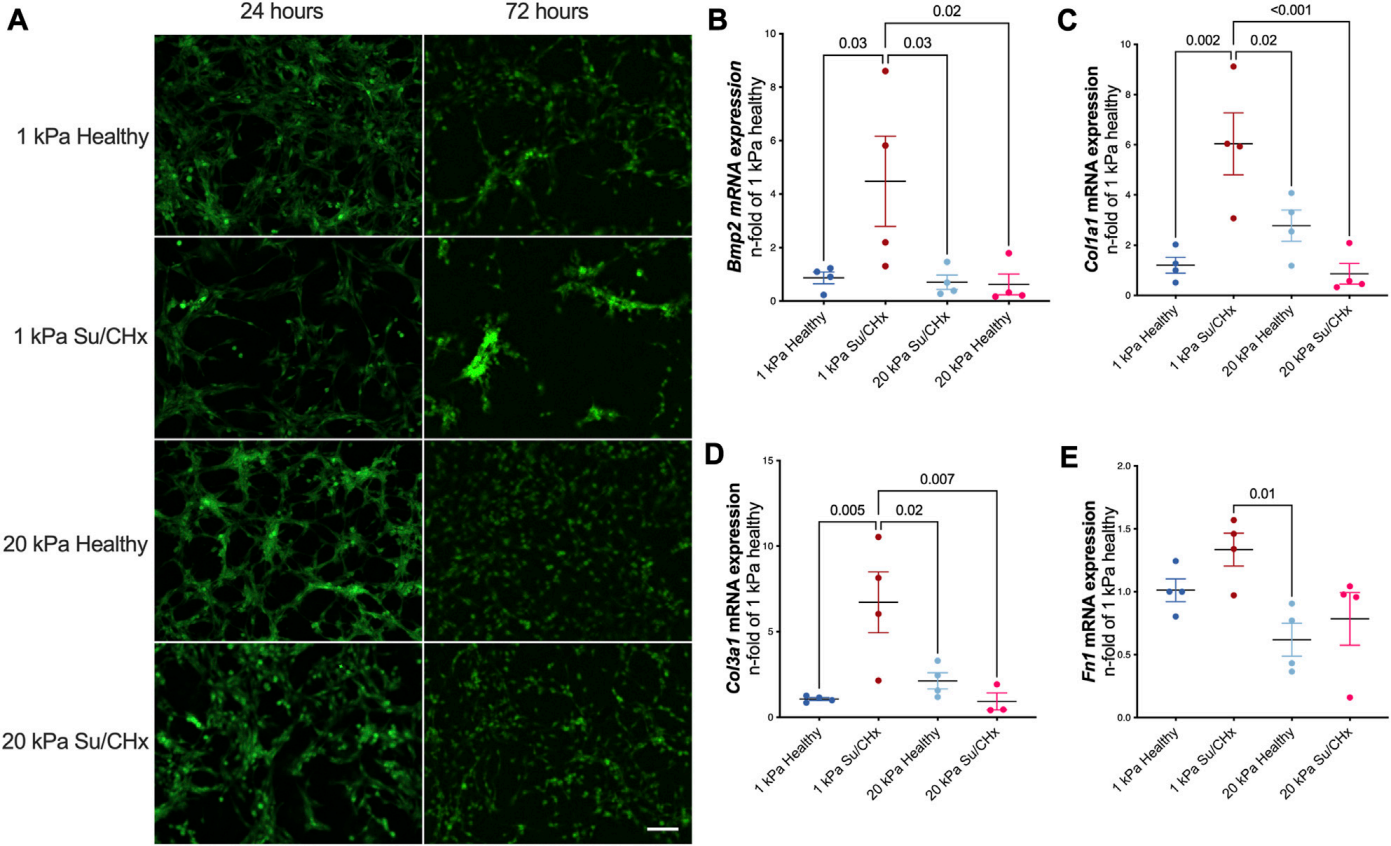
Diseased ECM changes tube formation and phenotype. **(A)** GFP positive CD117+ EC were plated on PAG hydrogels coated with healthy and diseased ECM. After 24 h tubules exist in all conditions. After 72 h, tubules are still present on 1 kPa healthy, cells cluster on 1 kPa Su/CHx. 20 kPa with both ECMs have increased proliferation though it appears there may be some tubulogenesis in the 20 kPa Su/CHx group. Scale 100 µm. **(B–E)** CD117+ ECs were added to ECM coated PAGs for 48 h. Changes are normalized to 1 kPa healthy for each gene. **(B)** *Bmp2*|Bone Morphogenic Protein-2; **(C)** *Col1α1*|Collagen type I α1; **(D)** *Col3α1*|Collagen type III α1; **(E)** *Fn1*|Fibronectin 1. Points indicate averaged technical replicates from *n* = 4 independent experiments. Statistical analysis was performed using a One-Way ANOVA with a Dunnett’s *post hoc* test. Data presented are mean ± SEM.

Cellular morphology demonstrated changes with diseased ECM (Figure 4A). At early timepoints cells attached and appeared to form networks, indicative of vessels. The density of vessels appeared to be greatest in the 1 kPa healthy condition but were not quantified. 72 h after plating, CD117+ ECs plated on Su/CHx ECM were clustering, indicative of both phenotypic changes (Neagu et al., 2010) and vessel instability (Li et al., 2012). These effects may be transitive because most vessels need support cells for stability (Newman et al., 2011; Chatterjee and Naik, 2012). However, CD117+ ECs have been shown to form functional vasculature from a single cell (Fang et al., 2012). On 20 kPa substrates, cells appeared to lose the networked appearance and become more like a monolayer.

#### 3.3.2 Su/CHx ECM alters CD117+ EC phenotype

ECM from Su/CHx lung tissue changes CD117+ ECs phenotype, primarily on healthy substrate stiffnesses. *Bmp2*, *Col1a1*, *Col3a1*, and *Fn1* mRNAs increased after 48 h on 1 kPa Su/CHx ECM (Figures 4B–E). *Bmp2* is expected to increase on softer substrates (Gilde et al., 2016), but we found similar expression levels when plated on healthy ECM of different stiffnesses and increased more on Su/CHx ECM. *Col1a1* and *Col3a1* increases initially on softer substrates with Su/CHx ECM and paradoxically decreases on 20 kPa Su/CHx substrates. We expect that signaling from the Su/CHx matrix indicates to cells that the substrate must be stiffer, increasing collagen production. However, on stiffer substrates, there is already signaling confirming the stiffness and with increased collagen content in the Su/CHx ECM, therefore the 20 kPa Su/CHx ECM group downregulates production of collagen.

## 4 Discussion

The aim of this study was to investigate whether the composition of the extracellular matrix (ECM) influences cell fate. To achieve this, we decellularized lungs from healthy and pulmonary arterial hypertension (PAH) rat models and digested the matrix to form an ECM coating solution. We then coated healthy and diseased ECM on healthy (1 kPa) and diseased (20 kPa) polyacrylamide substrates and observed the effects on CD117+ clonotype endothelial cells. We found that 24 h after plating, tube formation was similar in all conditions, but only healthy ECM on healthy stiffness retained the appearance of tube formation 72 h after plating. Supplementary Figure S1B shows quantification of the 72 h timepoint. We also observed changes in gene expression 48 h after plating, including pro-angiogenic BMP2 and ECM markers Col1α1 and Col3α1, which may indicate a dysregulated phenotype associated with aberrant angiogenesis in PAH.

Understanding the contribution of ECM composition is crucial since both substrate stiffness and decellularized lung matrix, which combines stiffness and diseased composition, have been shown to influence the disease state (Scarritt et al., 2014; Stenmark et al., 2016). In addition, individual lipids and ECM proteins have been implicated in the progression and resolution of PAH (Orr et al., 2006; Bu et al., 2008; Gairhe et al., 2016). Our work identified changes in many lipids between intact healthy and diseased tissues, and trends were largely retained upon decellularization, albeit likely considerably decreased after decellularization. We observed trends of increased sphingosine, sphingosine 1 phosphate, dihydrosphingosine 1, and ceramides being retained in ECM at increased levels from Su/CHx ECM that likely altered the cell behavior. Sphingosine 1 phosphate is important in maintaining endothelial cell-cell junctions (Maceyka and Spiegel, 2014) and promoting angiogenesis (Williams et al., 2015; Weigel et al., 2023). Ceramides can also promote barrier dysfunction and apoptosis in endothelium (McVey et al., 2021). Our findings point toward the importance of these lipids in cell-ECM signaling.

The protein changes we identified between decellularized and healthy ECM were largely consistent with other reports of proteomics changes in PAH (Suntharalingam et al., 2008; Huang et al., 2018). Notably, extracellular proteins found in significantly different quantities in the decellularized tissues include Fibrinogen α and Laminin-ß2, both of which have been shown to play a role in PAH or processes associated with PAH. Fibrinogen concentration is associated with PAH and linked to disease severity and decreased barrier integrity (Lominadze et al., 2010; Hennigs et al., 2014). We have previously shown integrin β5, capable of binding to the RGD sequences of ECM proteins, to have a variable role in PAH (Blanchard et al., 2022), which may be explained through different ECM protein signals. Laminin changes occur in vascular remodeling, and altered laminin content may indicate vascular instability (Lugassy et al., 1999). However, many of the identified proteins are intracellular proteins that could act as damage-associated molecular patterns (DAMPs), eliciting an immunogenic response, causing the cellular phenotype shifts we see. We expect a general increase in DAMPs in decellularized tissue, but balancing decellularization methodology to remove cell debris yet retain as much of the native ECM and composition as possible has been shown to be desirable (Badylak, 2014). By retaining the composition, we can begin to investigate the effects of composition on cell phenotypes.

CD117+ endothelial cells have been implicated in the progression of PAH, as demonstrated by previous studies (Farkas et al., 2014; Farkas et al., 2019; Bhagwani et al., 2020). We have previously shown a susceptibility of CD117+ ECs to Endothelial-to-mesenchymal transition (Bhagwani et al., 2020). In this study, we observed that CD117+ ECs expressed higher levels of Col1α1 and Col3α1 in response to diseased ECM on healthy stiffness, suggesting that early changes to the ECM composition may contribute to the aberrant angiogenesis found in PAH. Previous research has shown that hypoxia can increase Col1α1 and Col3α1 expression in cell culture after 7 days (Zhang et al., 2018), and our data supports the notion that these changes may result from early alterations to the ECM composition. Additionally, our previous work has shown that CD117+ ECs expressing high levels of BMP2 form occlusive lesions when exposed to hypoxia (Bhagwani et al., 2019). Therefore, the findings presented in this study may provide insight into an early mechanism for the aberrant angiogenesis observed in PAH.

Our study has some limitations, such as the 2D nature of the experiments, which may not reliably test composition and stiffness on a monolayer. The proliferation of the cells needs to be further examined to take into account the attachment and analysis of the clonality of the CD117+ ECs. The efficacy of chunk decellularization depends on detergent contact time and piece size, so smaller pieces and longer periods in contact with detergents may improve decellularization. However, we believe that our approach retains a good approximation of the ECM composition after decellularization that provides basis for using this method in further study.

In conclusion, we showed that ECM compositional differences in cellular fragments, structural ECM, and lipid families are retained in decellularized lung ECM from Su/CHx rats compared with healthy controls. We also demonstrated the feasibility of examining the ECM composition separated from the substrate stiffness by utilizing decellularized matrix from diseased and healthy rats coated onto stiffness-controlled polyacrylamide gels. Our work furthers the field by presenting methodology and relevant endothelial cell phenotype response to compositional changes in a common rat model of PAH. Future studies could investigate the role of single sphingolipids or individual ECM proteins in cellular signaling events in PAH, as well as test human cells, human PAH-derived ECM and stiffness changes to better understand the role of ECM composition independent of organization and innate stiffness.

## Data Availability

The original contributions presented in the study are included in the article/Supplementary Material, further inquiries can be directed to the corresponding author.

## Supplementary material

SUPPLEMENTAL FIGURE S1
**(A)** Su/CHx ECM does not affect CD117+ ECs proliferation on PAGs. CD117+ ECs plated on collagen, healthy matrix, and Su/CHx matrix have similar proliferation on different stiffnesses after 24 h. *n*=2. **(B)** Percent vessel coverage from an angiogenesis assay conducted following 72 h cultured on the varied conditions. Vessel percentage significantly decreases with Su/CHx ECM or increased stiffness. *n*=3.
