# Supplementary figures and images for: Using extracellular matrix derived from sugen-chronic hypoxia lung tissue to study pulmonary arterial hypertension
Source: Front Pharmacol. 2023 Sep 5;14:1192798. doi: 10.3389/fphar.2023.1192798 (PMC10507686; doi:10.3389/fphar.2023.1192798)

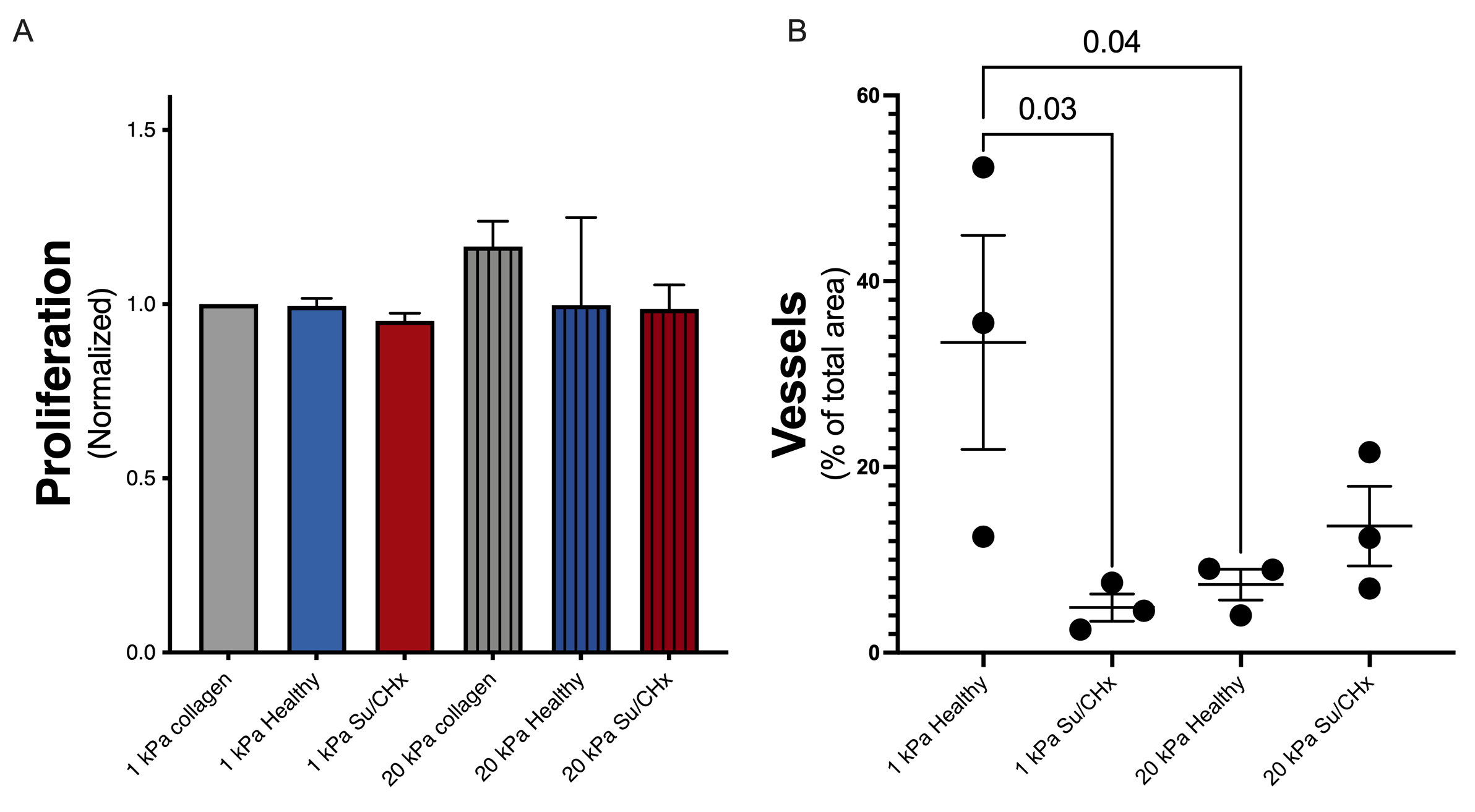

Supplement: Supplementary file 2 [file Image1.TIFF]
